# Discharge of treated Fukushima nuclear accident contaminated water: macroscopic and microscopic simulations

**DOI:** 10.1093/nsr/nwab209

**Published:** 2021-11-26

**Authors:** Yi Liu, Xue-Qing Guo, Sun-Wei Li, Jian-Min Zhang, Zhen-Zhong Hu

**Affiliations:** Institute for Ocean Engineering, Shenzhen International Graduate School, Tsinghua University, China; Institute for Ocean Engineering, Shenzhen International Graduate School, Tsinghua University, China; Institute for Ocean Engineering, Shenzhen International Graduate School, Tsinghua University, China; Institute for Ocean Engineering, Tsinghua University, China; Department of Hydraulic Engineering, Tsinghua University, China; Institute for Ocean Engineering, Shenzhen International Graduate School, Tsinghua University, China; Shenzhen Key Laboratory of Marine IntelliSense and Computation, Shenzhen International Graduate School, Tsinghua University, China

## Abstract

The diffusion process of the treated Fukushima nuclear accident contaminated water to be discharged into the Pacific Ocean from 2023 is analyzed by two analysis models from macroscopic and microscopic perspectives. Results show that the tritium will spread to the whole North Pacific in 1200 days, which is important to formulate global coping strategies.

On 26 August 2021, the Japanese Cabinet passed a bill to discharge treated Fukushima nuclear accident contaminated water into the Pacific Ocean to alleviate the problem of nuclear wastewater storage. The discharge of treated water, 1 km offshore, will begin in 2023. However, >60 radionuclides exist in the contaminated water, and the water in >70% tanks requires secondary treatments to meet discharge standards [1]. Large amounts of radionuclides can affect marine biological chains when inhaled by marine life and adversely influence marine fisheries and human health. Thus, identifying the diffusion process of radioactive water in oceans is critical, and helps predict possible effects on marine environments.

The diffusion of radioactive materials in oceans is a new branch of science [2]. After numerically simulating Fukushima radionuclides through interpolation [3], GEOMAR (Helmholtz Centre for Ocean Research Kiel) adopted the global ocean circulation model to estimate the long-term dispersion process of Cs-137 in Fukushima nuclear leakage [4]. In 2013, Lai *et al.* reproduced the coastal inundation and the initial spread of Cs-137 in the Fukushima earthquake and tsunami [5]. To obtain early warnings and decision support, studies have optimized diffusion models by considering the time-varying quality of the diffusion coefficients and the atmospheric stability. Recent investigations on Fukushima treated water discharge have provided simulations for four discharge scenarios [6]. However, the radionuclide diffusion law in oceans remains unclear. The ocean environment undergoes complex changes over a long time, which makes describing internal flow fields by governing equations difficult.

Therefore, the macroscopic and microscopic diffusions of nuclear pollutants in the ocean were analysed. Macroscopic and microscopic diffusion processes focus on the overall distribution of pollutants and behaviour of individual pollutants, respectively. Both these processes were categorized into several subprocesses. These subprocesses were individually analysed and then superimposed to obtain the overall diffusion simulation. For subprocess derivation, Fick's law [7] and Einstein's theory of mean square displacement [8] were adopted. Figure 1a presents the subprocesses of macroscopic and microscopic diffusion analyses and their relationships.

**Figure 1.**
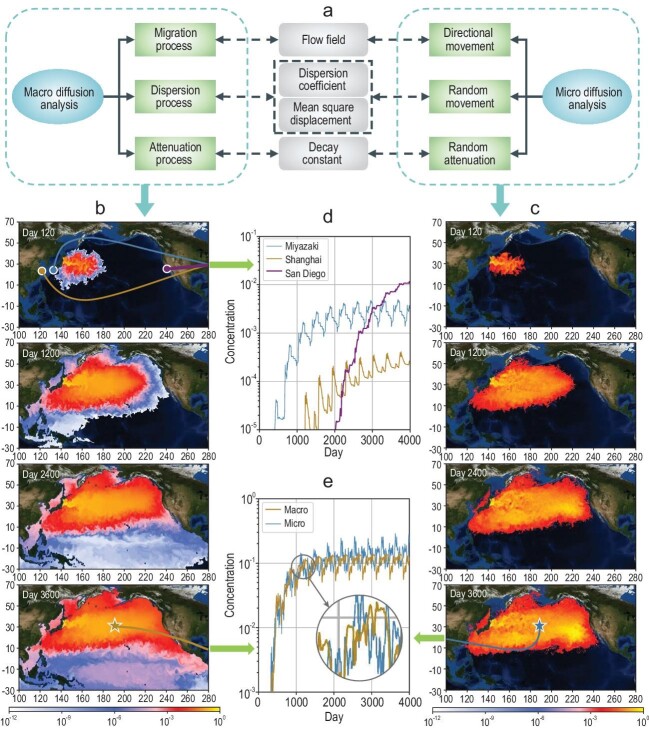
(a) Subprocesses of macroscopic and microscopic diffusion analyses and their relationships. Results of (b) macroscopic and (c) microscopic diffusion analyses. (d) Variations in the pollutant concentration in the waters near the three coastal cities. (e) Comparison of the pollutant concentration curves by macro and micro methods.

Tritium is the main pollutant in the treated water that Japan is planning to discharge. Thus, tritium diffusion was simulated with appropriate parameters. Figure 1b and c illustrate the results for 10 years for 1 unit relative concentration of ∼$0.29\,{\rm{Bq}}/{{\rm{m}}^3}$, according to the discharge plan. The simulations are based on the inference of small-scale diffusion analyses presented in the supplementary materials. The ocean surface current data are obtained from Earth & Space Research [9].

The macro simulation results revealed that in the early stages of pollutant discharge, the polluted area increases rapidly, reaching 30° of latitude × 40° of longitude within 120 days. Due to ocean currents, the pollutant diffusion speed is considerably higher in the latitude direction than the longitude direction. The high-concentration strip area remains near 35°N. After 1200 days, the pollutants arrive at the North American and Australian coasts to the east and south, respectively, and thus almost cover the entire North Pacific region. Then, these pollutants travel along the Panama Canal due to the equatorial current and rapidly spread over the South Pacific Ocean. Within 2400 days, along with diffusion into the Pacific Ocean, a small part of the pollutant spreads to the Indian Ocean through the waters north of Australia. After 3600 days, the pollutants occupy almost the entire Pacific Ocean. Although pollutant discharge occurs near the Japanese island, over time, water with high pollutant concentrations (yellow and red parts) moves eastward along the latitude of 35°N.

Figure 1b and d show three coastal cities near 30°N and the changes that occur in pollutant concentrations in their adjacent waters within 4000 days, respectively. Among these three cities, pollutants first appear near Miyazaki, followed by Shanghai and San Diego. This sequence is mainly determined by their distances from Fukushima. According to the trend of the three curves, the pollutant concentration in each region rapidly increases initially but then stabilizes. Although the pollutants reach San Diego last, the steady-state concentration of the pollutants in its adjacent waters is higher than that near the other two cities. This phenomenon results from the strong ocean current near Japan. Specifically, Fukushima is located at the confluence of Kuroshio (northward) and Oyashio (southward). Therefore, most pollutants do not migrate towards north and south along the land edges but spread eastward with the North Pacific west wind drift. This result indicates that in the early stage of treated water discharge, the impact of nuclear pollutants on the coastal waters of Asia should be focused. However, at a subsequent stage, because the pollutant concentration in the coastal waters adjacent to North America remains higher than that in most East Asian coasts, considering the effect of pollutants on North America is important.

A lower concentration occurs in the micro simulation than in the macro results because of the limited number of pollutant particles. Because the micro simulation provides the location of each pollutant particle, it supports pollutant-trajectory analyses. Figure 1e shows that the results of the two methods based on completely different physical and mathematical principles are consistent. Furthermore, our results are more specific than the results of GEOMAR [4] and Zhao *et al.* [6] because the actual continuous discharge plan, updated data, highly accurate models and parameters, and the Pacific and Indian Oceans are considered. GEOMAR and Zhao *et al.* considered a discharge duration of several weeks [4] or 10 years [6], whereas the up-to-date plan considered a duration of >30 years. However, previous simulation results also support the conclusion that ‘the pollutant concentration in the coastal waters adjacent to North America remains higher than that in most East Asian coasts’, which is proposed for the first time in this study. Thus, the results are feasible and reliable. In addition, the maximum background concentration of tritium along the Fukushima coast is ∼$290\,{\rm{Bq}}/{{\rm{m}}^3}$ [10]. The simulation results of the concentration increase remain important for quantitative prediction of long-term radionuclide diffusion, reasonable response to the discharge plan, subsequent experiments on environmental impacts and further studies on ecology sensitivity to radioactive substances.

## Supplementary Material

nwab209_Supplemental_Files
