# Supplementary material for: Discharge of treated Fukushima nuclear accident contaminated water: macroscopic and microscopic simulations
Source: Natl Sci Rev. 2021 Nov 26;9(1):nwab209. doi: 10.1093/nsr/nwab209 (PMC8776541; doi:10.1093/nsr/nwab209)
Supplement: nwab209_Supplemental_Files [file nwab209_supplemental_files.zip › Supplementary_Data_Clear.docx]

Supplementary Materials
for
“Discharge of treated Fukushima nuclear accident contaminated water: macroscopic and microscopic simulations”

Detailed Methods

Simulation Parameters

Extended Data Analysis

Figs. S1 to S13

Movies S1 to S4 (animations of the simulation results)

Detailed Methods

**1 Macroscopic diffusion analysis**

In the macroscopic diffusion analysis, the following design is adopted. The sea area is discretized into a series of small grids, and the pollutant concentration change of each grid within a time step is calculated. This process is then iterated, thereby obtaining the pollutant concentration of each grid at a specified moment. In the macro diffusion analysis, the diffusion process is approximately decomposed into three independent sub-processes, namely the migration, dispersion, and attenuation processes, as displayed in Figure S1. The migration process refers to the directional movement of pollutants with the ocean current, which only changes the location of pollutants but not the concentration. The dispersion process indicates the transport of pollutants from high-concentration areas to low-concentration areas because of the concentration gradient, which is mainly affected by phenomena such as molecular diffusion, turbulent diffusion under local turbulence, and dispersion due to uneven cross-sectional velocities. The attenuation process refers to the process of concentration reduction when the pollutants are transformed into other substances due to their own decomposition or decay.


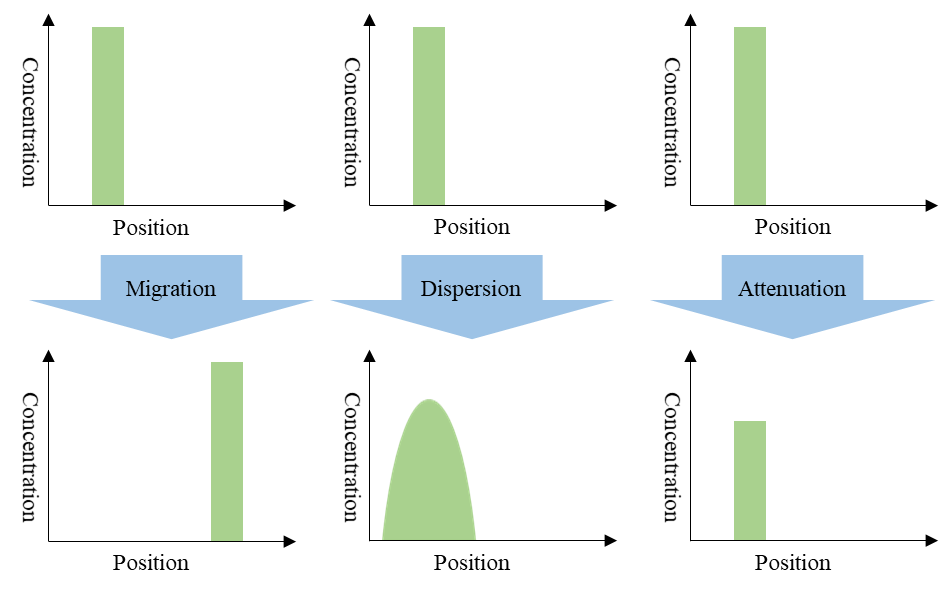


**Fig. S1. Migration, dispersion, and attenuation processes.** In the macroscopic diffusion analysis, the diffusion process is approximately decomposed into the three independent sub-processes.

**1.1 Dispersion process**

The key feature of the dispersion process is that the concentration change of pollutants is proportional to the concentration gradient. Their ratio, which is the dispersion coefficient, is related to the aforementioned molecular diffusion, turbulent diffusion, and dispersion. It can be expressed in the form of Fick’s law [7] as follows:

$J=-D\frac{\partial c}{\partial x}$ (1)

where $D$ is the dispersion coefficient, ${\partial c}/{\partial x}$ is the concentration gradient, and $J$ is the diffusion flux denoting the total amount of pollutants passing through a unit area in unit time.

Considering the two-dimensional dispersion problem, that is, the pollutants are evenly distributed along the height direction. First, the two-dimensional plane is uniformly divided into small grids. The side length of the grid along the $x$-direction is $\Delta x$, and the side length along the $y$-direction is $\Delta y$. Generally, the two-dimensional plane can be divided into squares such that $\Delta x=\Delta y$. The pollutant concentrations at the centre of two adjacent grids are $c_{1}$ and $c_{2}$ ($c_{1}\geq c_{2}$), and their difference is $c_{d}=c_{1}-c_{2}$, as displayed in Figure S2.


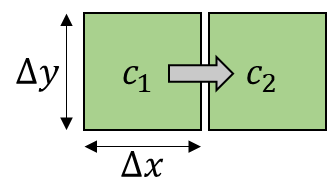


**Fig. S2. Two adjacent small grids.** The lengths of the two sides of the rectangle are $\Delta x$ and $\Delta y$. The pollutant concentrations at the centre of two adjacent grids are $c_{1}$ and $c_{2}$.

Assuming that the concentration gradient between the centre points of two grids is constant, the following expression can be applied:

$\frac{\partial c}{\partial x}=\frac{c_{d}}{\Delta x}$ (2)

In a short period of $\Delta t$, the concentrations of the two grids do not change considerably, and the concentration gradient remains unchanged. Subsequently, the total amount of pollutants passing through the contact surface of the two grids is expressed as follows:

$\Delta n=\left| J\times S\times\Delta t \right|=D\frac{c_{d}}{\Delta x}\times S\times\Delta t$ (3)

where $S$ is the area of the contact surface for the two grids. Assuming that the pollutants are evenly distributed along the height direction, and the height of the contact surface is $h$, then $S=h\times\Delta y$.

Before and after the pollutants pass through the contact surface, the concentration variations of the two grids are expressed as follows:

$\Delta c=\frac{\Delta n}{V}=\frac{D\frac{c_{d}}{\Delta x}\times h\times\Delta y\times\Delta t}{\Delta x\times\Delta y\times h}=\frac{Dc_{d}\Delta t}{{(\Delta x)}^{2}}$ (4)

In other words,

$\frac{\Delta c}{c_{d}}=D\frac{\Delta t}{{(\Delta x)}^{2}}$ (5)

The aforementioned formula reveals that when the dispersion coefficient $D$ remains unchanged, if the side length $\Delta x$ of the small grid along a certain direction and the simulation time step $\Delta t$ are given, ${\Delta c}/{c_{d}}$ becomes a constant expressed as follows:

$\frac{\Delta c}{c_{d}}=k=D\frac{\Delta t}{{(\Delta x)}^{2}}$ (6)

The value of $k$ represents the simulation speed of the dispersion process, and its significance can be understood through the following process. For any two adjacent grids, the difference in pollutant concentration at the centre points is $c_{d}$. After one simulation step, the pollutant concentration of the low-concentration grid increases by $kc_{d}$, whereas the pollutant concentration of the high-concentration grid decreases by $kc_{d}$. The larger $k$ is, the fewer simulation steps are required to reach the uniform concentration state. However, a significant change in the concentration gradient during one step of the simulation process (when the previous assumptions remain unchanged) corresponds to less accurate results. In terms of division of square grids, as $\Delta x=\Delta y$, the values along the two directions are the same. To simplify the problem, the default grid is considered a square in the subsequent analysis. In addition, a small square generally has four adjacent squares; thus, $k\leq1/{(4+1)}=0.2$ must be satisfied to ensure the convergence of the results. For three-dimensional dispersion problems, the critical value is $1/{(6+1)}=0.14$. Furthermore, $k$ indicates that the dispersion process is only related to the relative concentration and is unrelated with the absolute value of the concentration. The following is a simple example to illustrate the calculation of the dispersion process (Figure S3). The concentration of a central small square is 1 (relative value), and the concentrations of the surrounding squares are all 0; let $k=0.02$. After one simulation step, the concentration of the adjacent squares becomes $0+0.02\times\left( 1-0 \right)=0.02$, whereas that of the central small square becomes $1-0.02\times\left( 1-0 \right)\times4=0.92$.


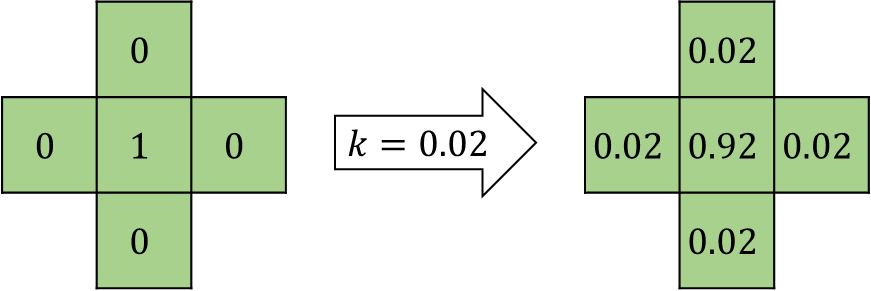


**Fig. S3. Example of macro diffusion simulation.** This is an example showing the changes of pollutant concentration within a time step when the simulation speed $k=0.02$.

**1.2 Migration process**

In the migration process, only the directional movement of pollutants with the current is considered. The basic assumption is that at the same time and location, the pollutants have the same migration direction and speed as the flow field of the ocean current. For the two-dimensional migration problem, the speed of the flow field at the centre of the small square is $v$, and its components along the two orthogonal directions are $v_{x}$ and $v_{y}$, respectively. After a short period of $\Delta t$, the fluid element at the centre of the small square advances by a distance of $v_{x}\Delta t$ and $v_{y}\Delta t$ along the $x$-direction and the $y$-direction, respectively, arriving at a new position. At this stage, the concentration at the new location after migration is equal to that at the centre of the small square before migration. Because this method only records the concentration of pollutants at the centre of each square, to keep the grid division of the sea area unchanged, it is necessary to set the moving distance to be an integral multiple of the side length $\Delta x$. Assuming $\left\langle a \right\rangle=\left\lfloor a+0.5 \right\rfloor$ (rounding), the number of squares advanced by the fluid element at the square centre can be recorded as $\left\langle{v_{x}\Delta t}/{\Delta x} \right\rangle$ and $\left\langle{v_{y}\Delta t}/{\Delta x} \right\rangle$ along the $x$-direction and $y$-direction, respectively. However, the rounding operation can lead to data distortion. For example, both $\left\langle0.5 \right\rangle$ and $\left\langle1.4 \right\rangle$ are equal to 1, but the contents expressed by 0.5 and 1.4 differ. To reduce the error caused by the rounding operation and retain as much velocity information as possible, $\left\langle{v_{x}\Delta t}/{\Delta x} \right\rangle$ and $\left\langle{v_{y}\Delta t}/{\Delta x} \right\rangle$ should be large or ${v\Delta t}/{\Delta x}$ should be far greater than 1.

Within a time step $\Delta t$, the pollutants disperse into the adjacent small squares, that is, the pollutants diffuse by one square in the dispersion process but move approximately a distance of $\left\langle{v\Delta t}/{\Delta x} \right\rangle$ squares in the migration process. If $\left\langle{v\Delta t}/{\Delta x} \right\rangle$ is greater than 1, the pollutants cannot diffuse upstream, resulting in an ‘empty space’ in pollution simulation, as indicated by the ‘?’ square in Figure S4.


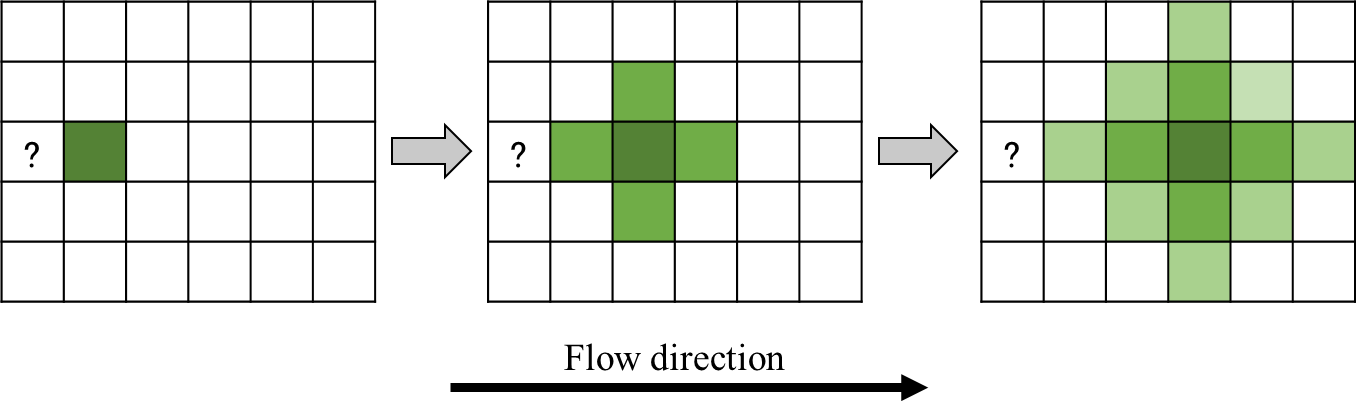


Fig. S4. ‘Empty space’ of pollution in the upstream direction. No matter how long it takes, the pollutants cannot diffuse to the ‘?’ square when $\boldsymbol{v\Delta t}/\boldsymbol{\Delta x}\boldsymbol{>1}$.

However, this result is in contradiction with the ${v\Delta t}/{\Delta x}\gg1$ requirement by the previous analysis. This problem can be solved by assuming different time steps for the two processes. Specifically, only when $\beta$ dispersion processes have been calculated, one migration process is calculated. Thus, the time steps of the dispersion and migration processes are changed to $\Delta t$ and $\beta\Delta t$, respectively, where $\beta$ is a positive integer. The dispersion process can result in diffusion by a distance of $\beta$ small squares in a period of $\beta\Delta t$, during which the pollutants can move a distance of $\left\langle{\beta v\Delta t}/{\Delta x} \right\rangle$ small squares approximately, and only the following condition is required:

$\left\{ \begin{aligned} \frac{\beta v\Delta t}{\Delta x}<\beta\\ \frac{\beta v\Delta t}{\Delta x}\gg1 \end{aligned} \right. => \frac{1}{\beta}\ll\frac{v\Delta t}{\Delta x}<1$ (7)

Typically, the maximum velocity $v_{max}$ of the ocean current can be used instead of$v$ in the aforementioned formula.

The meaning of $\beta$ can be further explained by the following process. In fact, after a time step $\Delta T$, the pollutant diffuses not only to adjacent squares, but also to many other squares, and the diffusion flux can be directly obtained by spatial distribution of point source diffusion. However, different boundary conditions will lead to the difference of spatial distribution, resulting in a large amount of computations. Another relatively simple solution is to divide $\Delta T$ to many (let’s say $N$) small $\Delta t$, and the distribution calculated after $N$ steps of $\Delta t$ can be considered as the spatial distribution after $\Delta T$. This solution is much easier to implement and allows faster simulation.

Another potential problem is that because of fluid deformability, the centres of multiple small squares may move inside the same grid after migration. As displayed in Figure S5(A), after migration, the centres of squares *a* and *c* moved into square *d*, whereas no square centre moved to square *b*. Thus, square *d* has multiple concentration inputs, whereas square *b* has no concentration input in the calculation of the migration process. Therefore, the reverse derivation method is used to calculate the migration process. Specifically, for the centre of each small square to be solved, the position of the fluid element before $\beta\Delta t$ is calculated, and the pollutant concentration at this position is the pollutant concentration at the centre of the small square after migration. As displayed in Figure S5(B), for solving squares *b* and *d*, a unique point corresponding to each centre is determined. This method ensures that each square has a unique concentration input, which satisfies the analysis and calculation requirement of the migration process. However, this approach may lead to a loss of concentration data (the concentration data of square *a* is used repeatedly, whereas the concentration of square *c* is not used at all).


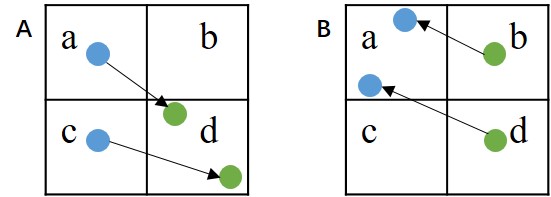


**Fig. S5. Forward and reverse derivations of the migration process.** (A) Forward derivation; (B) Reverse derivation.

**1.3 Attenuation process**

For the problem of nuclear pollution, the attenuation process mainly involves the decay of radioactive elements. The process of decay is only related to the element and not with environmental conditions, such as temperature and humidity, which can be specifically expressed using the following equation:

$\frac{dc}{dt}=-\lambda c$ (8)

where $c$ is the concentration of the radioactive material, and $\lambda$ is the decay constant. Thus, the concentration of the radioactive material becomes $e^{-\lambda\Delta t}$ of the original value after an arbitrary time of $\Delta t$. The relationship between the decay constant and the half-life $T_{1/2}$ is expressed as follows:

$\lambda=\frac{\ln2}{T_{1/2}}$ (9)

For a single pollutant discharge, the concentrations of all squares are multiplied by $e^{-\lambda\Delta t}$ each time in the calculation of the attenuation process. However, the calculation of the dispersion process is only related to the relative concentration, and the calculation of the migration process only involves the ocean current data. Therefore, the attenuation process in each period need not be considered, and only the difference $t$ between the start time and end time of the diffusion process and $e^{-\lambda t}$ as the multiplication of the calculated result should suffice.

**2 Microscopic diffusion analysis**

The variation in the pollutant concentration is equivalent to the change in the quantity of pollutant particles per unit volume. Therefore, the diffusion process can also be analysed from the perspective of pollutant particles. Micro diffusion analysis involves dividing the pollutants into numerous particles and then calculating the movement of each particle in a short time interval. Through this iterative process, the concentration of pollutants is represented by the number of particles in each region. Figure S6 displays the particle distribution in a certain scenario. By dividing the plane into various regions and counting the particles in each region, the relative pollutant concentration in each region can be estimated. Similarly, in micro diffusion analysis, the change in pollutant particles can be categorised into three independent processes, namely the directional movement, random movement, and random attenuation, which correspond to the migration process, dispersion process, and attenuation process in macro diffusion analysis. The three processes in the micro diffusion analysis are discussed individually in the following section.


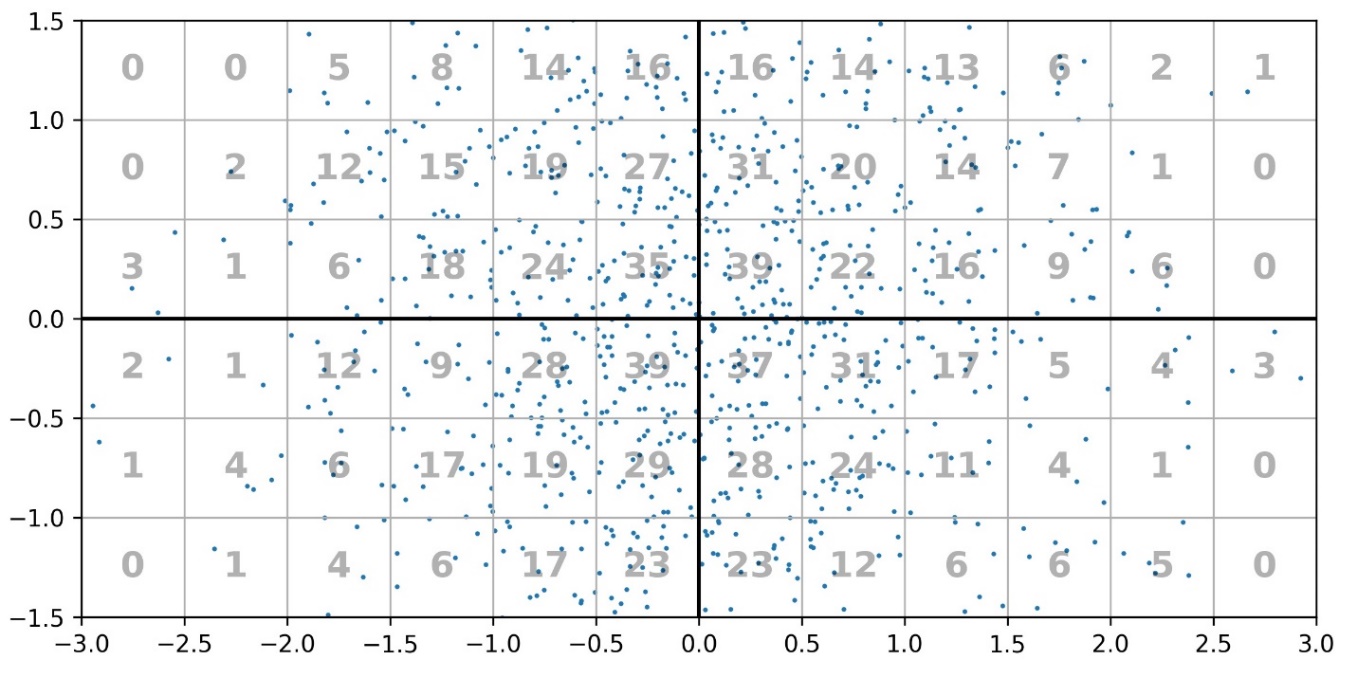


**Fig. S6. Example of particle distribution count.** The relative pollutant concentration in each region can be estimated by the quantity of pollutant particles.

**2.1 Random movement**

Random movement is a nonstop, irregular motion, such as the Brownian motion. Although the random movement of an individual particle is irregular, the random movement of numerous particles satisfies statistical rules. For instance, macroscopically, pollutants move from high-concentration to low-concentration regions. In mathematical analogy, random movement is similar to random walk. Einstein analysed the random walk problem and concluded that the mean square displacement of particles is proportional to the dispersion coefficient and time interval [8]. For one-dimensional problems, this relationship can be expressed as follows:

$\bar{x^{2}}=2D\Delta t$ (10)

where $\bar{x^{2}}$ is the mean square displacement of the one-dimensional random walk, that is, the average of the squared particle displacement within a time interval of $\Delta t$. For two-dimensional problems, the square of the actual displacement equals the sum of the squares of the displacements in two orthogonal directions, which are generally independent and identically distributed. Therefore, we have the following expression:

$\bar{r^{2}}=2\bar{x^{2}}=4D\Delta t$ (11)

From the perspective of probability, the two-dimensional random walks in the two directions are independent of each other and follow the same 0-mean normal distribution. Thus, the displacement magnitude of the two-dimensional random walk follows Rayleigh distribution, and the displacement direction is uniformly distributed; the two are independent of each other. Assuming the displacement magnitude and direction of particles are expressed by $r$ and $\theta$, respectively, and the corresponding probability density functions are expressed as $f\left( r \right)$ and $g\left( \theta\right)$, respectively, then we have the following expression:

$\left\{ \begin{aligned} f\left( r \right)=\frac{r}{\sigma^{2}}e^{-\frac{r^{2}}{2\sigma^{2}}} r\geq0 \\ g\left( \theta\right)=\frac{1}{2\pi} 0\leq\theta<2\pi\end{aligned} \right.$ (12)

Therefore, the expected value of the squared displacement magnitude is expressed as follows:

$E\left( r^{2} \right)=\int_{0}^{\infty} r^{2}\times\frac{r}{\sigma^{2}}e^{-\frac{r^{2}}{2\sigma^{2}}}dr=2\sigma^{2}$ (13)

According to the definition of expectation, $E\left( r^{2} \right)=\bar{r^{2}}$, we have the following expression:

$2\sigma^{2}=4D\Delta t => \sigma=\sqrt{2D\Delta t}$ (14)

Therefore, for the random movement in micro diffusion analysis, the displacement magnitudes of pollutant particles within $\Delta t$ follow a Rayleigh distribution with a parameter of $\sqrt{2D\Delta t}$, where $D$ is the macroscopically measured dispersion coefficient. When calculating the displacement caused by random movement, the displacement of each pollutant particle can be obtained with the programme that generates random numbers. The displacement direction angle $\theta$ is generated according to uniform distribution, whereas the displacement magnitude $r$ is produced according to Rayleigh distribution.

**2.2 Directional movement**

The analysis of the directional movement of pollutant particles is easier than that of the microscopic migration process. For the two-dimensional problem, the speed of ocean current where the pollutant particles are located is $v$, and its components along the two orthogonal directions are $v_{x}$ and $v_{y}$, respectively. In a short period of $\Delta t$, the displacement of the pollutant particles due to directional movement is $v\Delta t$, and the components along the two orthogonal directions are $v_{x}\Delta t$ and $v_{y}\Delta t$. This method can be used to accurately record the position of each pollutant particle. Therefore, the approximation of $v_{x}\Delta t$ and $v_{y}\Delta t$ is not necessary.

Furthermore, the premise for calculating the directional motion displacement is that the magnitude and direction of velocity for the pollutant particle change negligibly within one time step; hence, $\Delta t$ should not be too large. The displacements of random movement and directional movement can subsequently be added in a vectorial manner, obtaining the actual displacement of pollutant particles within $\Delta t$.

**2.3 Random attenuation**

The random attenuation of nuclear pollution mainly considers the decay of pollutants. From the microscopic perspective, the decay constant represents the probability of decay for a single particle in unit time. Therefore, random attenuation can be realised as follows: within one time step $\Delta t$, any pollutant particle has a probability of $1-e^{-\lambda\Delta t}$ to disappear. When $\lambda\Delta t$ is a small value, the vanishing probability can be approximated by $\lambda\Delta t$. Similarly, if we only require the distribution of pollutants at the end time and no pollutants are added during the simulation, considering random attenuation in each time step is not necessary. Instead, the calculated result can be multiplied with $e^{-\lambda t}$ according to the difference $t$ between the start time and end time of the diffusion process.

**Simulation Parameters**

**1 Fundamentals**

Fukushima is located at 37°N, 141°E. According to the report by related Japanese agencies, the discharge is expected to last for 30 to 40 years, and the main pollutants in the treated water that Japan plans to discharge is tritium. Because the half-life of tritium is 12.43 years, the decay constant of tritium can be calculated to be $1.768\times{10}^{-9}s^{-1}$. After the discharge, the radioactive water mostly diffused in the Pacific Ocean; thus, only the water from 95°E to 295°E and from 80°S to 80°N was considered in the analysis. In subsequent analysis and simulation, the lengths for one degree of longitude and latitude are approximated to be $100 km$. Moreover, the deepest part of the Pacific Ocean is only approximately $10 km$, which is considerably smaller than its length and width. Therefore, in this simulation, the problem was considered a two-dimensional diffusion. The dispersion coefficient in zonal and meridional directions are assumed to be constant $D=300{m^{2}}/s$.

Japan's plan to discharge Fukushima treated water into the sea involves a maximum daily discharge of 500 tonnes of contaminated water before purification, and the tritium content in the contaminated water is approximately 730,000 becquerels per litre. In the simulation, it is assumed that the treated water is discharged continuously and periodically; hence, pollutants are added to the initial polluted area every five days. The initial pollutants are supposed to be uniformly distributed in a 0.8° × 0.8° square area centred at 142°E, 37°N. In addition, because the calculation process of diffusion analysis is not related to the absolute concentration, the concentration of pollutants added each time is assumed to be one unit when calculating the concentration at each position at different moments. Or, the aforementioned data can be used for rough estimation; for example, the total amount of tritium discharged every five days is approximately $1.825\times{10}^{12} \mathrm{Bq}$, assuming that the average depth at which the pollutants arrive is $1000 m$. One unit of pollutant concentration can be approximately considered to be $0.29\mathrm{Bq}/{m^{3}}$.

The ocean surface current analysis real-time (OSCAR) data set contains the current velocity at ocean surfaces from 80°S to 80°N, with an accuracy of 1/3° and a time interval of 5 days [9]. In this study, the data for 2020 in the OSCAR data set was selected to generate the ocean current data within the simulation time by cycling the data. According to a brief data analysis, the maximum sea surface current speed was $v_{max}=4.75m/s$ (Figure S7).


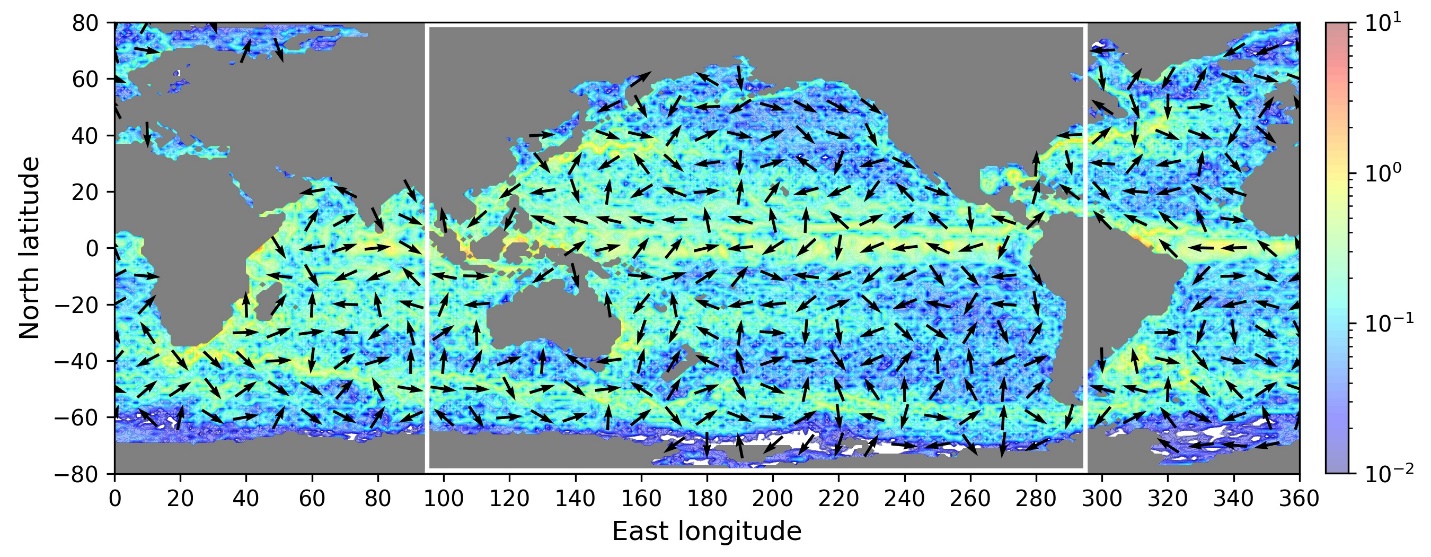


**Fig. S7. Global ocean currents on January 1st, 2020.** The grey parts are land, while the other parts are the ocean, and the white border represents the boundaries of the simulation. The unit of color bar is $m/s$.

**2 Parameters in macro diffusion simulation**

To apply the macro diffusion analysis method for simulation, first, the sea area to be analysed is divided according to the longitude and latitude, and the side length of each small square is set to be 0.2°, corresponding to a length of $\Delta x=20 km$. According to the aforementioned analysis, the simulation time step $\Delta t$ of the dispersion process should satisfy the following expression:

$\frac{v_{max}\Delta t}{\Delta x}<1 => \Delta t<\frac{20000m}{4.75m/s}=4211s$ (15)

Assuming $\Delta t=3600 s$, that is, 1 h, the simulation speed $k$ of the dispersion process is calculated as follows:

$k=D\frac{\Delta t}{{(\Delta x)}^{2}}=300{m^{2}}/s\times\frac{3600s}{{(20000m)}^{2}}=0.0027<0.2$ (16)

This value of $\Delta t$ can satisfy the convergence requirement in the dispersion process, and the simulation accuracy is high when the value of $k$ is small. Next, the time step coefficient, $\beta$, in the migration process should be determined, which must satisfy the following expression:

$\frac{1}{\beta}\ll\frac{v_{max}\Delta t}{\Delta x} => \beta\gg\frac{\Delta x}{v_{max}\Delta t}=\frac{20000m}{4.75m/s\times3600s}=1.17$ (17)

Let $\beta=120$, then the time step of the migration process is 5 days. When simulating the migration process, the displacement should not be calculated by $v\beta\Delta t$ in one single step but should be solved by the sum of $v\Delta t$ in $\beta$ times. Here, $v$ varies with the change in position (the cumulative displacement), thus avoiding result distortion caused by large time steps in the migration process.

Because the accuracy of the ocean current data of OSCAR is only 1/3°, it is difficult to satisfy the computation requirements. Therefore, the bilinear interpolation algorithm, a commonly used method to refine grid data, was adopted to encrypt the OSCAR grid to estimate the points inside the grid with the following function:

$v\left( x,y \right)=axy+bx+cy+d$ (18)

where coefficients $a$, $b$, $c$, and $d$ are obtained by substituting the data of the four corners of the grid, and generally the grids exhibit distinct coefficients. With this approach, the current velocity at any given location can be calculated to satisfy the calculation requirements of the migration process.

**3 Parameters in micro diffusion simulation**

The main parameter in the micro diffusion analysis is the time step $\Delta t$. Involving the consideration of both calculation accuracy and cost, the simulation takes 4 h as the time step, that is, $\Delta t=4\times3600s=14400s$.

When estimating the concentration, calculating the number of particles contained in a region is critical. Therefore, dividing the ocean area into small squares is crucial. If the side length of the squares is configured to be 0.2°, then the initial pollution area will also occupy 16 squares. Notably, the grid division is only for counting the pollutant particles, and it does not affect the calculation in the simulation process, which differs from the macro diffusion analysis. In each square, a 5-day discharge interval is set, and 1000 pollution particles are evenly injected each time. If random attenuation is not considered, there will be 12.8 million pollution particles on day 4000. For the simulation results, the number of pollution particles in each square is divided by 1000 to obtain the relative concentration of pollutants. As the number of particles is an integer, the resolution of the actual concentration that can be obtained is ${10}^{-3}$. Therefore, only those concentrations with a relative value in the range of $\left[ {10}^{-3}, 1 \right]$ are considered in the simulation results.

**4 Simulations on a small scale**

The previous simulations are carried out based on the assumption of “average depth”, which is reasonable according to the fine three-dimensional analysis on a small temporal and spatial scale described in detail below.

Specifically, the Etopo1 dataset and the HYCOM GOFS3.1 dataset were adopted as topographic data and three-dimensional ocean current data, respectively. Considering that the vertical dispersion effect in the ocean is relatively weak, the vertical dispersion coefficient is set to $1/{25}$ of the horizontal dispersion coefficient. There are three simulations of different ranges: 0.2° of latitude × 0.2° of longitude (small range), 2° of latitude × 2° of longitude (medium range) and 20° of latitude × 50° of longitude (large range). The depth ranges of the latter two are both 0 m to 5000 m, while that of the former is 0 m to 100 m.

The small range simulation results are demonstrated in Figure S8. The concentration of pollutants is distributed in a circular pattern along the horizontal direction, indicating that the dispersion process plays a dominant role at the early stage of pollutant diffusion. It also reveals that the pollutant spreads rapidly at this stage, reaching an area with radius of 1° in a few hours. On the one hand, the effect of dispersion becomes more significant than that of migration as the scale decreases. On the other hand, the low current velocity near the shore further weakens the migration effect. As shown in Figures S8 (B) and (C), the pollutants are evenly distributed from the sea surface to the seabed due to the shallow water. Note that the concentration mentioned here refers to the concentration increment. It can be revealed that the background concentration is about $0.3\mathrm{Bq}/{m^{3}}$ as indicated within the area of 1.3 km in Figure S8 (A), which is only about 1 % of the relative concentration, warning that about 100 times increment of tritium concentration in local area around the discharging point.


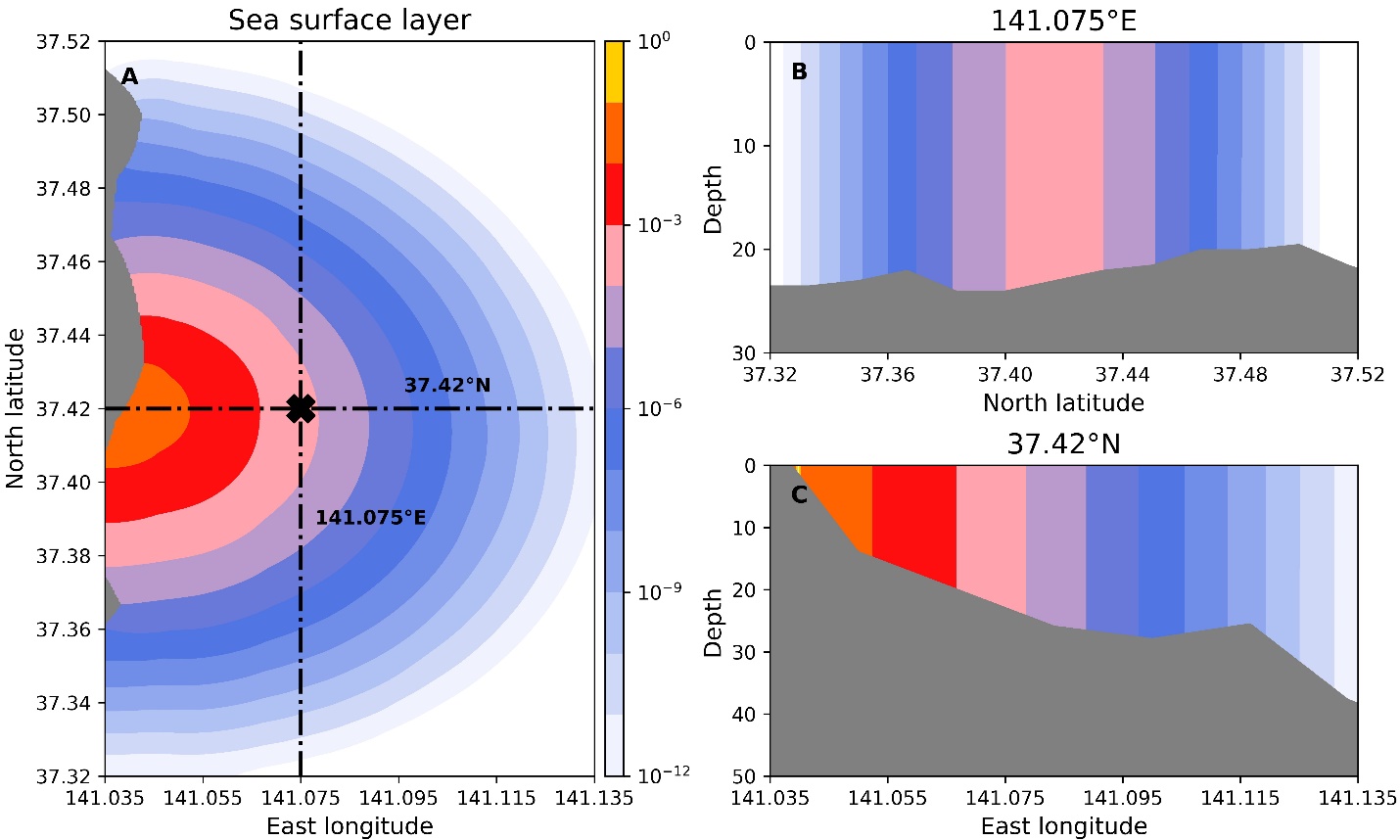


**Fig. S8. Concentration distribution at 200th minute (based on** **small range simulation).** (A) The horizontal concentration distribution of tritium in the sea surface layer. (B) The concentration distribution of tritium in the section of 141.075° E. (C) The concentration distribution of tritium in the section of 37.42° N. The grey parts are land (for A) and seabed (for B and C), while the other parts are the ocean. The unit of color bar is a unit relative concentration (about $31.68\mathrm{Bq}/{m^{3}}$), and the unit of depth is m.

The medium range simulation results are demonstrated in Figure S9. Compared with the small range simulation, the effect of migration is significantly enhanced. The overall pollutant distribution in the sea surface is approximately elliptic, and the central position of the pollution area has deviated from the discharging point. The pollutant concentration still appears to be evenly distributed along the vertical direction. The background concentration corresponds roughly to 0.1 relative concentration.


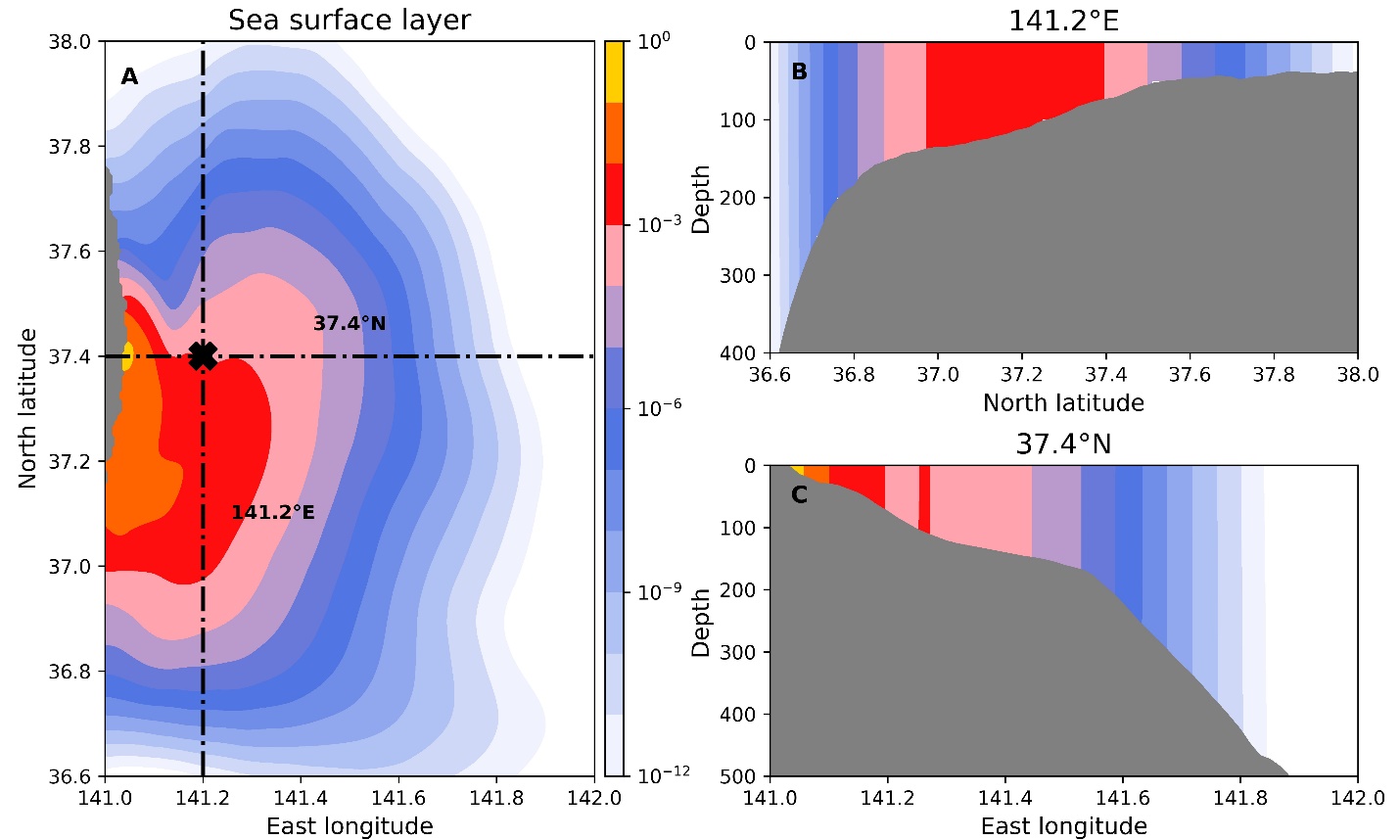


**Fig. S9. Concentration distribution at 200th hour (based on medium range simulation).** (A) The horizontal concentration distribution of tritium in the sea surface layer. (B) The concentration distribution of tritium in the section of 141.2° E. (C) The concentration distribution of tritium in the section of 37.4° N. The grey parts are land (for A) and seabed (for B and C), while the other parts are the ocean. The unit of color bar is a unit relative concentration (about $3.042\mathrm{Bq}/{m^{3}}$), and the unit of depth is m.

The large range simulation results are demonstrated in Figure S10. The migration has obviously played a dominant role at this range, and the diffusion velocity of pollutants along the latitude is significantly higher than that along the longitude. Due to the increase of grid size, the pollutant concentration of 1 unit is about 1/10 of the background concentration. Along the vertical direction, although the uniform distribution is not satisfied everywhere, the variation of pollutant concentration is ignorable.


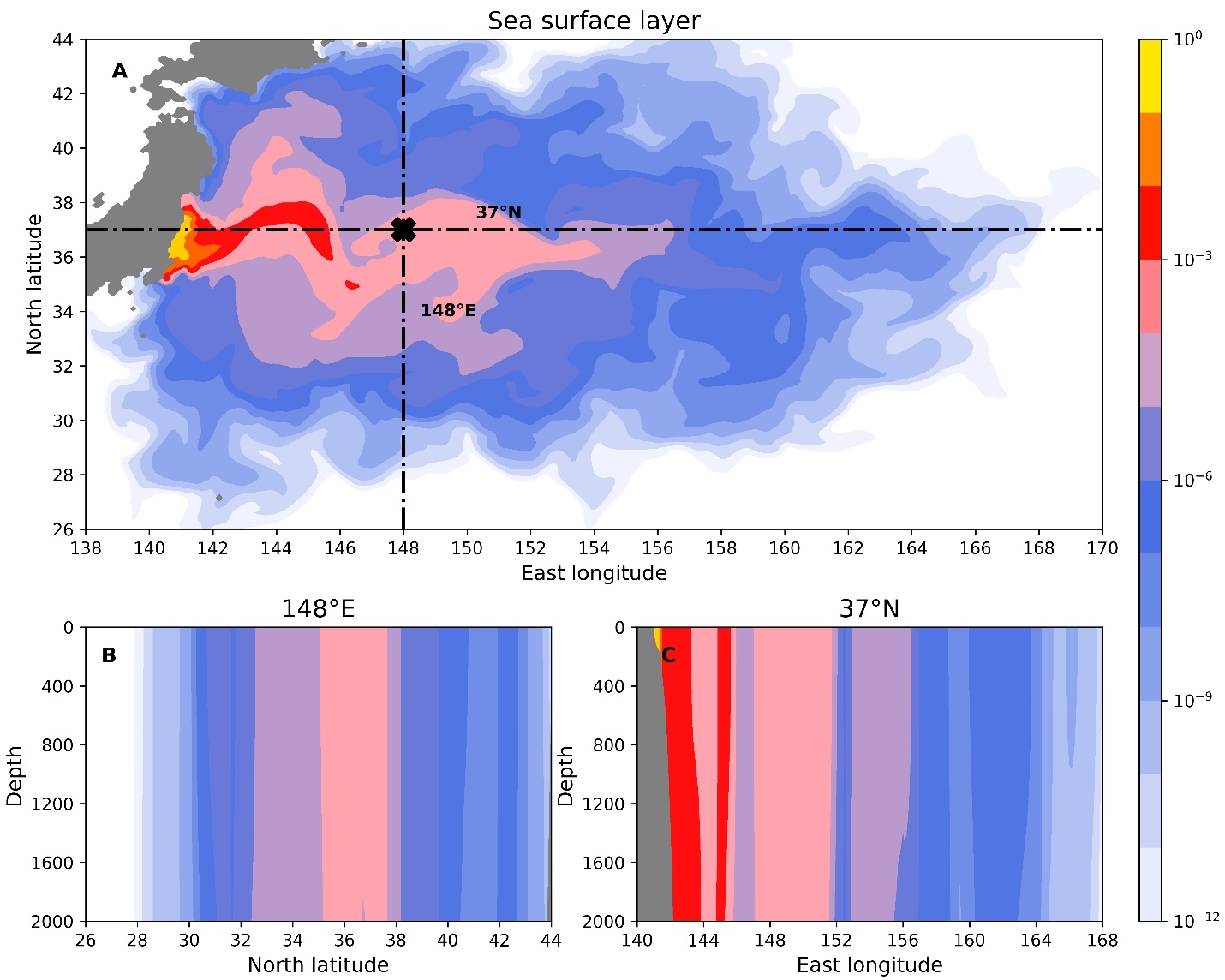


**Fig. S10. Concentration distribution on day 100 (based on large range simulation).** (A) The horizontal concentration distribution of tritium in the ocean surface layer. (B) The concentration distribution of tritium in the section of 148° E. (C) The concentration distribution of tritium in the section of 37° N. The grey parts are land (for A) and seabed (for B and C), while the other parts are the ocean. The unit of color bar is a unit relative concentration (about 30.42 Bq/m3), and the unit of depth is m.

The maximum duration of the above simulations is 100 days, while the discharge duration planned by the Japanese government is about 30-40 years. Over such a long duration, pollutants spread far more widely (as shown in the results of Figure 1 in the main text), and distribution is essential to predict their impacts on the surrounding area. Because the scale of the Pacific Ocean in the horizontal direction is much bigger than that in the vertical direction, it is necessary to divide a large number of grids with high precision to carry out reasonable three-dimensional simulation, which requires huge computational resources. The results of the above three simulations have proved that this division is unnecessary, because the pollutant is approximately evenly distributed in the vertical direction.

**Extended Data Analysis**

Another function of the micro analysis method is determining the trajectory of pollutant particles and analysing the diffusion path of pollutants. For example, for certain three pollutant particles arriving at the coastal waters in the simulation results, their motion trajectories are obtained with a sampling interval of 400 days and displayed in Figure S8. These trajectories reveal that most pollutants in the waters off the coast of the Americas arrive there by crossing the Pacific Ocean. When special attention is required for pollutants in a certain location, the trajectories of the pollutant particles can be analysed to obtain the main source regions and paths of the pollutants.


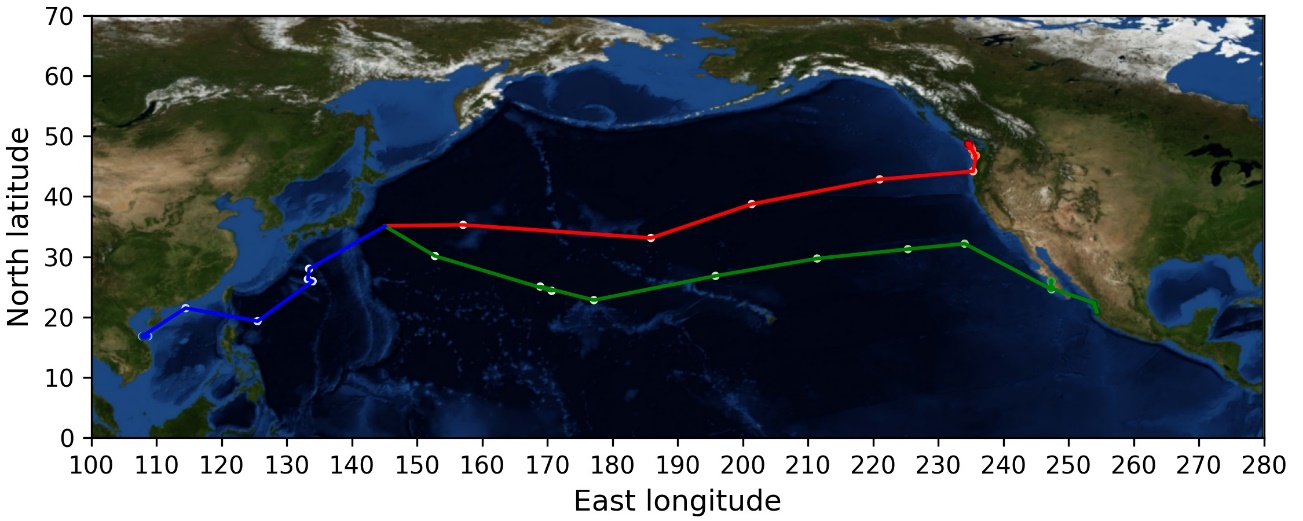


**Fig. S11. Trajectories of some pollutant particles.** The motion trajectories of three pollutant particles arriving at the coastal waters in the micro simulation results.

More comparison between simulation results obtained by the two methods are displayed in Figure S9.


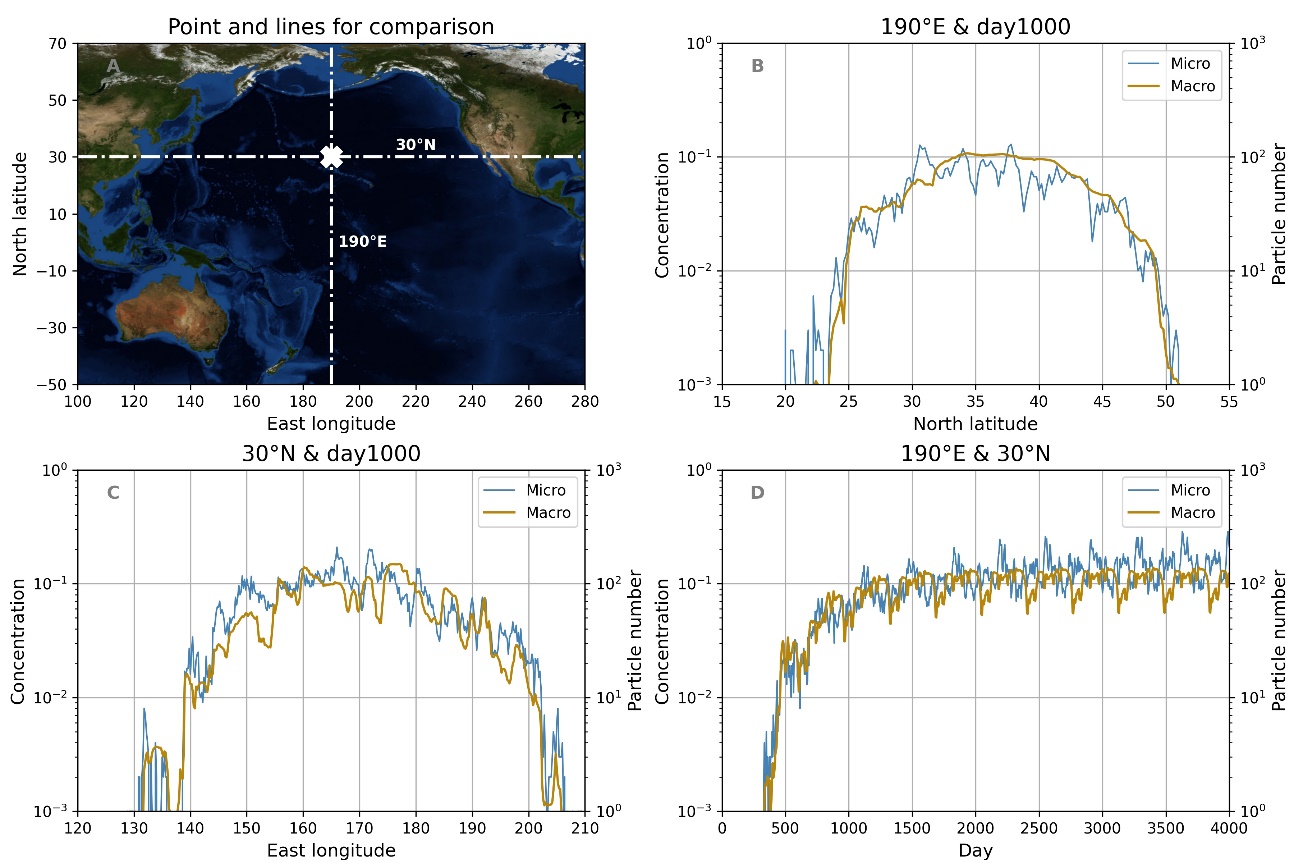


**Fig. S12. Comparison of macro and micro simulation results.** (A) Point and lines for comparison; (B) Variation of pollutant concentrations with latitude; (C) Variation of pollutant concentrations with longitude; (D) Variation of pollutant concentrations with time.

As for the diffusion processes of other types of radionuclides, in addition to their diffusion characteristics, the change of half-life should be considered. For example, the concentration distributions of Cs-134 and Sr-89 on day 1200 obtained by macro analysis are displayed in Figure S10. On day 1200, the concentration distribution of Cs-134 is almost the same as that of tritium, whereas the relative concentration of Sr-89 is considerably lower than that of tritium, as the half-life of Cs-134 is 2.06 years, whereas the half-life of Sr-89 is only 50.5 days.


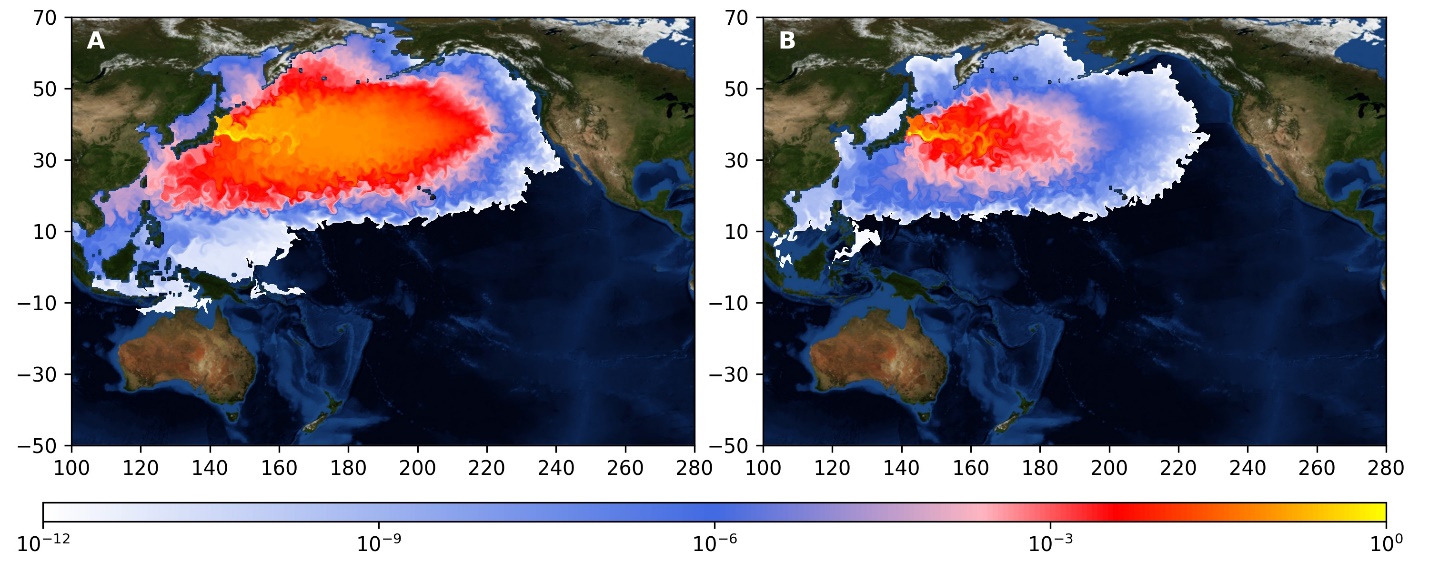


**Fig. S13. Concentration distributions of some other radionuclides on day 1200.** (A) Concentration distribution of Cs-134 on day 1200; (B) Concentration distribution of Sr-89 on day 1200. The unit of color bar is a unit relative concentration.

Movie S1.

Macro simulation of tritium.

Movie S2.

Micro simulation of tritium.

Movie S3.

Macro simulation of Cs-134.

Movie S4.

Macro simulation of Sr-89.
